# Clinical Assessment of Anti-Viral CD8+ T Cell Immune Monitoring Using QuantiFERON-CMV® Assay to Identify High Risk Allogeneic Hematopoietic Stem Cell Transplant Patients with CMV Infection Complications

**DOI:** 10.1371/journal.pone.0074744

**Published:** 2013-10-11

**Authors:** Siok-Keen Tey, Glen A. Kennedy, Deborah Cromer, Miles P. Davenport, Susan Walker, Linda I. Jones, Tania Crough, Simon T. Durrant, James A. Morton, Jason P. Butler, Ashish K. Misra, Geoffrey R. Hill, Rajiv Khanna

**Affiliations:** 1 Australian Centre for Vaccine Development and Tumour Immunology Laboratory, Queensland Institute of Medical Research, Herston, Queensland, Australia; 2 Department of Haematology and Bone Marrow Transplantation, Royal Brisbane and Women's Hospital, Herston, Queensland, Australia; 3 Bone Marrow Transplant Laboratory, Queensland Institute of Medical Research, Herston, Queensland, Australia; 4 School of Medicine, University of Queensland, Herston, Queensland, Australia; 5 Complex Systems in Biology Group, Centre for Vascular Research, University of New South Wales, Kensington, New South Wales, Australia; Beth Israel Deaconess Medical Center, Harvard Medical School, United States of America

## Abstract

The reconstitution of anti-viral cellular immunity following hematopoietic stem cell transplantation (HSCT) is crucial in preventing cytomegalovirus (CMV)-associated complications. Thus immunological monitoring has emerged as an important tool to better target pre-emptive anti-viral therapies. However, traditional laboratory-based assays are too cumbersome and complicated to implement in a clinical setting. Here we conducted a prospective study of a new whole blood assay (referred to as QuantiFERON-CMV®) to determine the clinical utility of measuring CMV-specific CD8+ T-cell responses as a prognostic tool. Forty-one evaluable allogeneic HSCT recipients underwent weekly immunological monitoring from day 21 post-transplant and of these 21 (51.2%) showed CMV reactivation and 29 (70.7%) developed acute graft-versus-host disease (GvHD). Patients with acute GvHD (grade≥2) within 6 weeks of transplant showed delayed reconstitution of CMV-specific T-cell immunity (p = 0.013) and a higher risk of CMV viremia (p = 0.026). The median time to stable CMV-specific immune reconstitution was 59 days and the incidence of CMV reactivation was lower in patients who developed this than those who did not (27% versus 65%; p = 0.031). Furthermore, a failure to reconstitute CMV-specific immunity soon after the onset of CMV viraemia was associated with higher peak viral loads (5685 copies/ml versus 875 copies/ml; p = 0.002). Hence, QuantiFERON-CMV® testing in the week following CMV viremia can be useful in identifying HSCT recipients at risk of complicated reactivation.

## Introduction

Allogeneic hematopoietic stem cell transplantation (HSCT) is an important treatment modality for a number of malignant and non-malignant hematological diseases. It is, however, associated with severe immune impairment and cytomegalovirus (CMV) reactivation is a significant and predictable complication [1], [2]. Clinical CMV disease, once established and clinically apparent, is associated with significant morbidity and can be fatal. Regular virological surveillance coupled with pre-emptive antiviral therapy upon subclinical reactivation is effective in preventing clinical disease and is standard practice [3], [4]. However, not all subclinical reactivation requires antiviral treatment: spontaneous viral clearance can occur in a proportion of patients who have evidence of CMV-specific immunity [5], [6], [7]. Conversely, a lack of CMV-specific immunity is associated with increased risks of recurrent or persistent CMV reactivation and CMV disease [6], [8], [9], [10], [11]. There is, therefore, a growing interest in the use of immunological monitoring to adjust treatment according to individual risk [5], [7], [12], [13], [14]. Hypothetically, patients with evidence of CMV-specific immunity may be observed without pre-emptive therapy, may tolerate higher viral loads in the absence of antiviral therapy without risk of disease, or may be managed with shorter courses of antiviral drugs. In contrast, patients with poor CMV-specific immunity may benefit from closer virological monitoring, a lower threshold for pre-emptive treatment, and longer treatment courses [2], [3], [6]. Importantly, poor immune reconstitution may be a useful laboratory criterion to select patients that are best treated with adoptive cellular therapy.

Traditional immunological assays are relatively complex and the effective implementation of immune monitoring in a routine clinical setting will require the use of an assay that can be readily performed in a routine diagnostic laboratory. Ideally, the test should use existing laboratory techniques, be easy to standardize with clear positive and negative cut-offs, be applicable to patients with diverse HLA alleles and, if possible, measure T cell function rather than numbers. A whole blood interferon-γ (IFN-γ) assay, known as QuantiFERON-CMV®, fulfils most of these criteria [15]. It is an ELISA-based functional assay that uses the same technology as a commonly used diagnostic test for *Mycobacterium tuberculosis* [16]. It directly measures the amount of IFN-γ secreted per volume of blood without the need for separate T cell enumeration. It includes 23 CMV peptide epitopes from diverse HLA alleles that together cover >95% of the general population [15]. It has been found to be useful in solid organ transplantation where it could predict the likelihood of spontaneous clearance of low level viraemia [17], [18], [19], [20], [21] and the risk of CMV disease following the cessation of valganciclovir prophylaxis [19]. It is CE (*Conformité Européenne*) marked for *in vitro* diagnostic use in Europe. Its potential application in allogeneic HSCT is not well-studied. The aim of this prospective observational study was to investigate the functional characteristics of the QuantiFERON-CMV® assay in allogeneic HSCT recipients and its relationship with CMV reactivation and disease.

## Methods

### Study subjects

This study was approved by the human ethics committees of the Royal Brisbane and Women's Hospital and the Queensland Institute of Medical Research (RBWH HREC reference number 2006/192. QIMR reference number P992). Patients undergoing allogeneic HSCT between 1 March 2007 and 1 March 2010 were eligible for inclusion and written informed consent was obtained from all participants. Patients with markers of active hepatitis B and hepatitis C were excluded. Myeloablative conditioning regimen consisted of cyclophosphamide 60 mg/kg/day for two days and 12 Gy of fractionated total body irradiation given over three days. Reduced intensity conditioning regimen consisted of fludarabine 25 mg/m^2^/day for 5 days and melphalan 120 mg/m^2^ for 1 day. Non-myeloablative conditioning regimen consisted of fludarabine 30 mg/m^2^ for 3 days and 2 Gy total body irradiation. All patients received a T-replete bone marrow or G-CSF-mobilised peripheral blood stem cell graft and none had *in vivo* T cell depletion. Graft-versus-host disease (GVHD) prophylaxis consisted of cyclosporine A and four doses of methotrexate given on days 1, 3, 6 and 11. Patients with contraindications to methotrexate received mycophenolate mofetil instead. Patients who were CMV seropositive and/or had a CMV seropositive donor received high dose acyclovir (500 mg/m^2^ intravenously 3 times daily) from day −5 to day +28 or until discharge, followed by valaciclovir 500 mg twice daily until day +100. Patients with plasma CMV DNAemia ≥600 copies/ml on virological surveillance were pre-emptively treated with intravenous ganciclovir at 5 mg/kg twice daily for induction, usually for 14 days, followed by maintenance at 5 mg/kg once daily until the plasma CMV DNA became undetectable (<600 copies/ml) on two consecutive tests. Patients with inadequate response or significant toxicity from ganciclovir were treated with foscarnet. Patients without venous access were sometimes treated with valganciclovir at 900 mg twice daily for induction and 900 mg once daily for maintenance. All drugs were dose adjusted for renal impairment.

### Virological monitoring

CMV DNA quantitation was performed on plasma samples at least once a week from day 0 to day +100. Monitoring beyond day 100 was dependent on the risk of late CMV reactivation as determined by the treating physician. The samples were batched and the assay was performed twice a week. Between March 2007 and March 2010, CMV DNA quantitation was performed with COBAS Amplicator CMV Monitor Test (Roche Diagnostics, Basel, Switzerland) after DNA extraction with MagNAPure LC Total Nucleic Acid Isolation Kit (Roche Applied Science, Penzberg, Germany). Results were linear from 600 to 100,000 copies/ml. From April 2010 onwards, CMV DNA quantitation was performed with the Qiagen Artus CMV kit (Qiagen, Doncaster, VIC) on ABI7500 platform (Applied Biosystems, Mulgrave, VIC) following DNA extraction with the Qiagen Symphony SP automated system. The limit of detection was 57 copies/ml and the results were linear to 5.5×10^6^copies/ml. CMV reactivation is defined as the detection of CMV DNA at ≥600 copies/ml. Sub-threshold CMV DNAemia is defined as the detection of CMV DNA at <600 copies/ml. Assay read-outs showing sub-threshold CMV DNAemia were recorded in the diagnostic laboratory log but not reported to the treating clinicians: both sub-threshold CMV DNAemia and undetectable CMV DNA were reported as ‘CMV DNA <600copies/ml’ in the formal laboratory report.

### Immunological monitoring

Immunological monitoring was performed with QuantiFERON-CMV® assay (Cellestis, Carnegie, VIC, Australia) which measured the amount of CMV-specific IFN-γ secretion in 1 ml of whole blood. The CMV peptide pool comprised 23 peptides derived predominantly from CMV pp65 and IE1, but also included epitopes from pp50, IE2 and gB. The HLA alleles represented were HLA-A1, A2, A3, A11, A23, A24, A26, B7, B8, B27, B35, B40/60, B41, B44, B52, B57 and B58. The peptides were at 2 µg each, resulting in a final peptide concentration of 2 µg/ml/peptide [22]. Peripheral blood was obtained at weekly intervals between 3 and 14 weeks after transplantation and at 6 and 12 months. The assay was performed according to the manufacturer's instruction. Briefly, 1 ml of heparinised whole blood was pipetted into each of three QuantiFERON-CMV® tubes: CMV peptide pool (‘CMV’), negative control (‘nil’), and positive control containing phytohemagglutinin (‘mitogen’). The tubes were incubated at 37°C for 15 to 24 hours after which the plasma was collected and stored at −80°C. Plasma IFN-γ levels were measured using standard ELISA technique and the concentration calculated from a log(e)-log(e) standard curve using software provided by the manufacturer. The samples were assayed neat. Repeat assay was performed at 1∶5 or 1∶10 dilution if the QuantiFERON-CMV® result was greater than the maximum standard. The interpretation of IFN-γ response, as recommended by the manufacturer, was as follows: ‘Positive’ if [CMV – nil] ≥0.2 IU/ml, ‘Negative’ if [CMV – nil] <0.2 IU/ml and [mitogen-nil] ≥0.5 IU/ml, and ‘Indeterminate’ if [CMV – nil] <0.2 IU/ml and [mitogen-nil] <0.5 IU/ml.

### Statistical analysis

The incidence of CMV reactivation and pre-emptive antiviral treatment were estimated using cumulative incidence estimates and differences between groups were calculated using log-rank test. Differences in categorical variables between two groups were calculated by Fisher's exact test. Differences in means of two groups were calculated by two-tailed *t* test. Viral loads were converted to a logarithmic scale to obtain a normal distribution and differences were therefore expressed in geometric mean. All reported *p* values are two-sided.

## Results

### Patient characteristics

Forty-six patients were prospectively recruited: four did not provide any study samples because of early death (3) or change of treating institution (1), and an additional patient was subsequently excluded because of active hepatitis C infection. The characteristics of the remaining 41 patients are summarised in Table 1.

**Table 1:** Characteristics of study population.

Median age, years (range)	51 (18–66)
Sex	23 Males, 18 Females
Median follow-up after transplant, days (range)	362 (47–671)
Diagnosis	
Acute leukemia	31
Chronic myeloid leukemia	2
Myelodysplastic syndrome	4
Myelofibrosis	2
Non-Hodgkin's lymphoma	1
Hemophagocytic lymphohistiocytosis	1
Preparative regimen	
Cyclophosphamide/total body irradiation	21
Fludarabine/melphalan	19
Fludarabine/total body irradiation	1
Donor source	
HLA-matched sibling	19
Matched unrelated donor	20
Mismatched unrelated (A, B or DR) donor	2
Stem cell source	
Bone marrow	3
Peripheral blood stem cells	37
Both	1
CMV donor/recipient serostatus	
Positive/Positive (D+R+)	14
Negative/Positive (D−R+)	24
Positive/Negative (D+R−)	3
Acute GVHD, any	29
Grade II-IV	22
Number of deaths, total	13
Death from relapse	8
Non-relapse death	5
GVHD contributory	3
Infection contributory	4
CMV contributory*	1
Graft rejection	1

* Multipathogen pneumonia with evidence of CMV pneumonitis.

### CMV reactivation and disease

A total of 37 episodes of CMV reactivation (DNAemia ≥600 copies/ml) were observed in 21 patients: 25 were early (within 100 days post transplant) and 12 were late (beyond 100 days post transplant); 16 patients had early reactivation, one had late reactivation, and 4 had both early and late reactivation. Eight patients had more than one episode of reactivation (range 2 to 6). Pre-emptive treatment was given to 34 out of 37 episodes. Three episodes were untreated: 1 patient was terminally ill from bacterial sepsis and died before antiviral treatment was instituted and 2 patients cleared the CMV DNAemia without antiviral treatment; neither had recurrent reactivation. In addition, 6 patients had a total of 8 episodes of sub-threshold CMV DNAemia (<600 copies/ml) which resolved without antiviral treatment. Biopsy-proven CMV disease was present in 3 patients and contributed to the death of 1 patient.

### QuantiFERON-CMV® analysis

A total of 495 QuantiFERON-CMV® samples were collected from 41 patients; the median number of samples per patient was 13 (range 2–14). A total of 232 samples (47%) were interpreted as QuantiFERON-CMV® positive (≥0.2 IU/ml), 74 (15%) as QuantiFERON-CMV negative (<0.2 IU/ml and mitogen ≥0.5 IU/ml) and 189 (38%) as indeterminate (CMV <0.2 IU/ml and mitogen <0.5 IU/ml). Both the QuantiFERON-CMV® and mitogen results significantly correlated with the lymphocyte count. The mean lymphocyte count was 1.86±0.09×10^9^/L (mean±SD) for samples with QuantiFERON-CMV ≥0.2 IU/ml and 0.90±0.04×10^9^/L for samples with QuantiFERON-CMV® <0.2 IU/ml (p<0.001). Similarly, the mean lymphocyte count was 1.75±0.08×10^9^/L for samples with Mitogen ≥0.5 IU/ml and 0.91±0.05×10^9^/L for samples with Mitogen <0.5 IU/ml (p<0.001). As a result, 76 of 92 samples (83%) collected at lymphocyte count <0.5×10^9^/L were considered ‘indeterminate’ as compared to 113 of 403 samples (28%) collected at lymphocyte counts ≥0.5×10^9^/L (p = 0.0001). Of note, samples collected at higher lymphocyte counts were more likely than samples collected at lower lymphocyte counts to be QuantiFERON-CMV® ‘negative’ (17% versus 8%, p = 0.034) because there were fewer ‘indeterminate’ results.

### Immune reconstitution and its relationship with CMV reactivation

**Figure 1:**
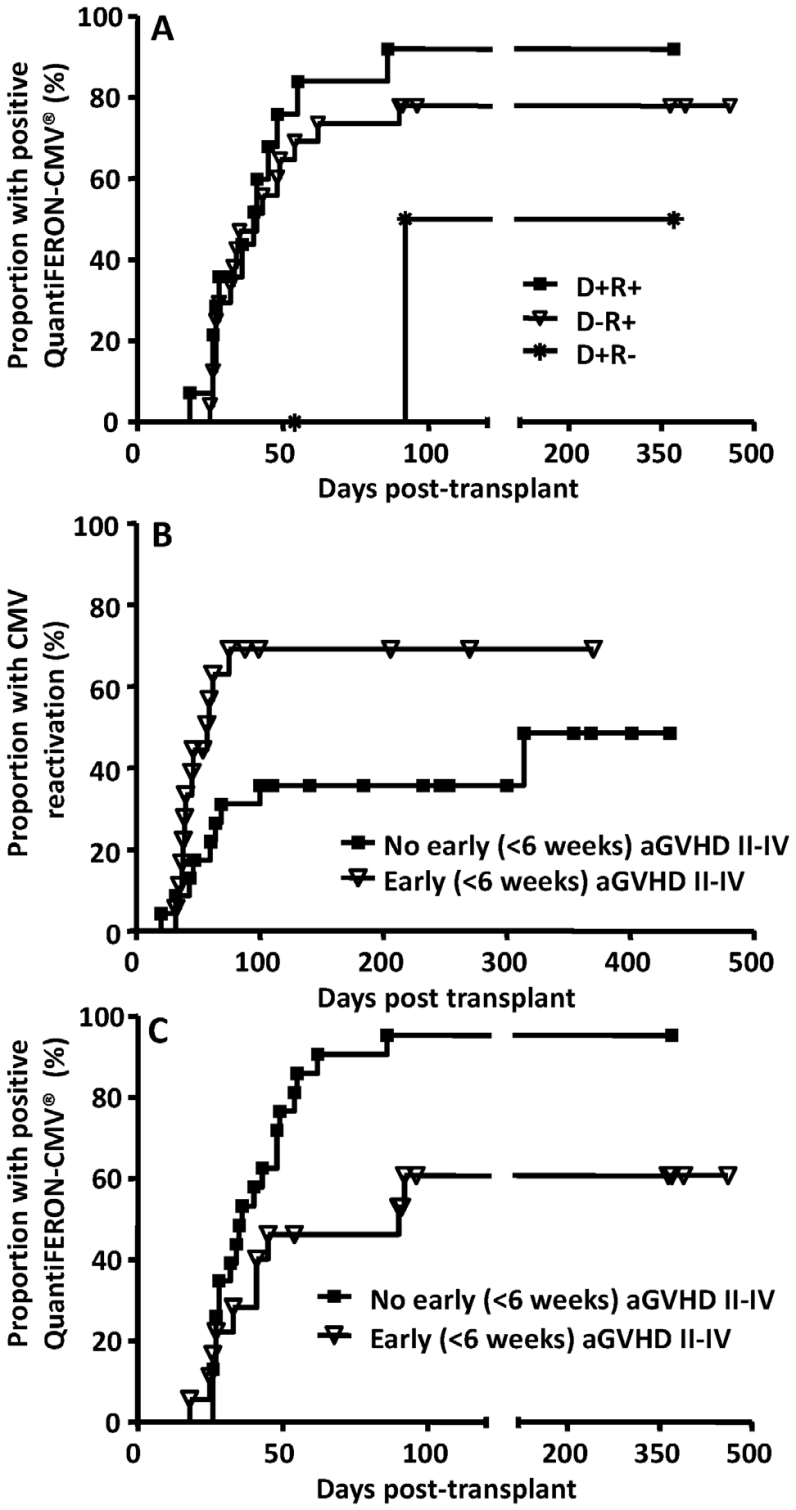
Cumulative incidence of CMV-specific CD8+ T cell immune reconstitution and CMV reactivation in HSCT recipients. Panel A: Reconstitution of CMV-specific T cell immunity according to donor and recipient CMV-serostatus. Panel B: CMV reactivation according to the development of GVHD grade ≥II within 6 weeks post transplant (*p* = 0.026, log-rank test). Panel C: Reconstitution of CMV-specific T cell immunity according to the development of GVHD grade ≥II within 6 weeks post transplant (*p* = 0.013, log-rank test).

Positive QuantiFERON-CMV® results developed in 31 of 41 patients at a median of 35 (range 18 to 92) days after transplantation. The time to CMV-specific immune reconstitution was similar in CMV-seropositive recipients irrespective of the donor serostatus (Fig. 1A); there were too few CMV-seronegative recipients for analysis. Patients who developed acute GVHD grade II–IV within 6 weeks post transplant were more likely to have CMV reactivation (69% versus 36%; p = 0.026) (Fig. 1B). Furthermore, these patients also had delayed reconstitution of CMV-specific CD8+ T cell immunity as assessed by QuantiFERON-CMV® assay (p = 0.013, log-rank test) (Fig. 1C).

**Figure 2:**
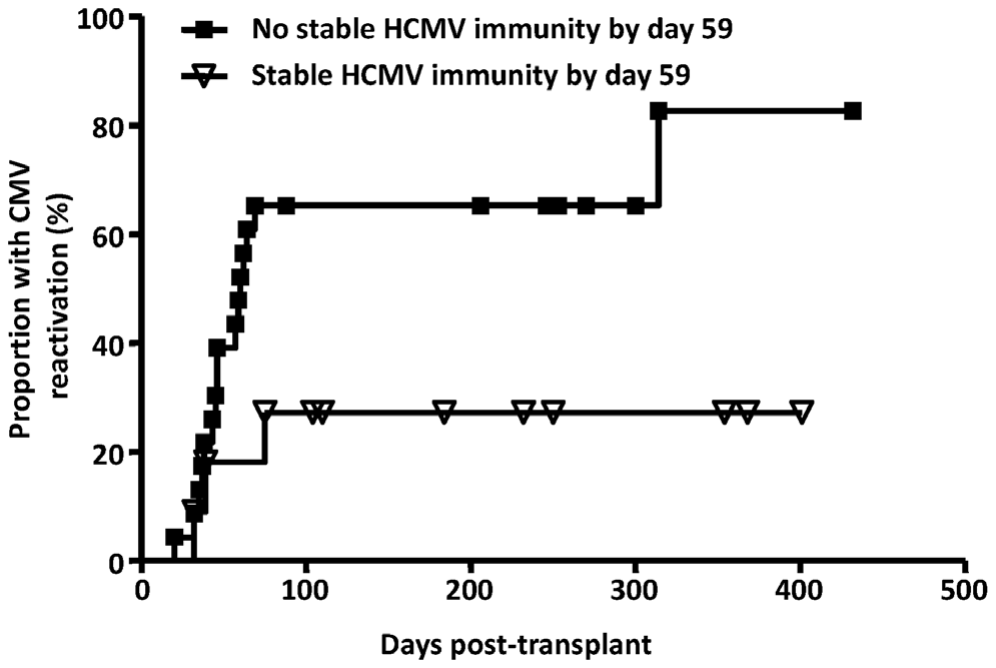
Relationship between stable CMV-specific CD8+ T cell immune reconstitution and CMV reactivation. Data presented in this graph shows the cumulative incidence of CMV reactivation according to the development of stable CMV-specific CD8+ T cell immune reconstitution by day 59.

Immune reconstitution after transplant is highly dynamic: 21 out of the 31 patients with a single positive QuantiFERON-CMV® reading subsequently had one or more negative or indeterminate results. There was no difference in the lymphocyte count at the time of first appearance and disappearance of positive QuantiFERON-CMV® reading (mean 1.08×10^9^/L versus 1.30×10^9^/L) but of the 21 patients, 15 were on corticosteroids at the time of loss of initial positive QuantiFERON-CMV® reading. Amongst CMV-seropositive recipients, those with and without early CMV reactivation had similar rates of CMV-specific immune reconstitution (data not shown). Over time, the immune response became more sustained. Stable immune reconstitution, here defined as persistent positive Quantiferon-CMV® results on weekly testing until at least day 90, was observed in 22 patients. The median time to stable CMV-specific immune reconstitution in these patients was 59 days. Patients who achieved stable immune reconstitution by day 59 had a lower rate of CMV reactivation compared to those who did not (27% versus 65%, p = 0.031) (Fig. 2). Importantly, of the 11 patients who achieved stable immune reconstitution by day 59, none developed CMV disease. In comparison, of the 23 patients who did not achieve stable immune reconstitution by day 59, 3 developed biopsy-proven CMV disease: one recurrent colitis (day 145 and day 234), one enteritis (day 168) and one pneumonitis (day 82).

**Figure 3:**
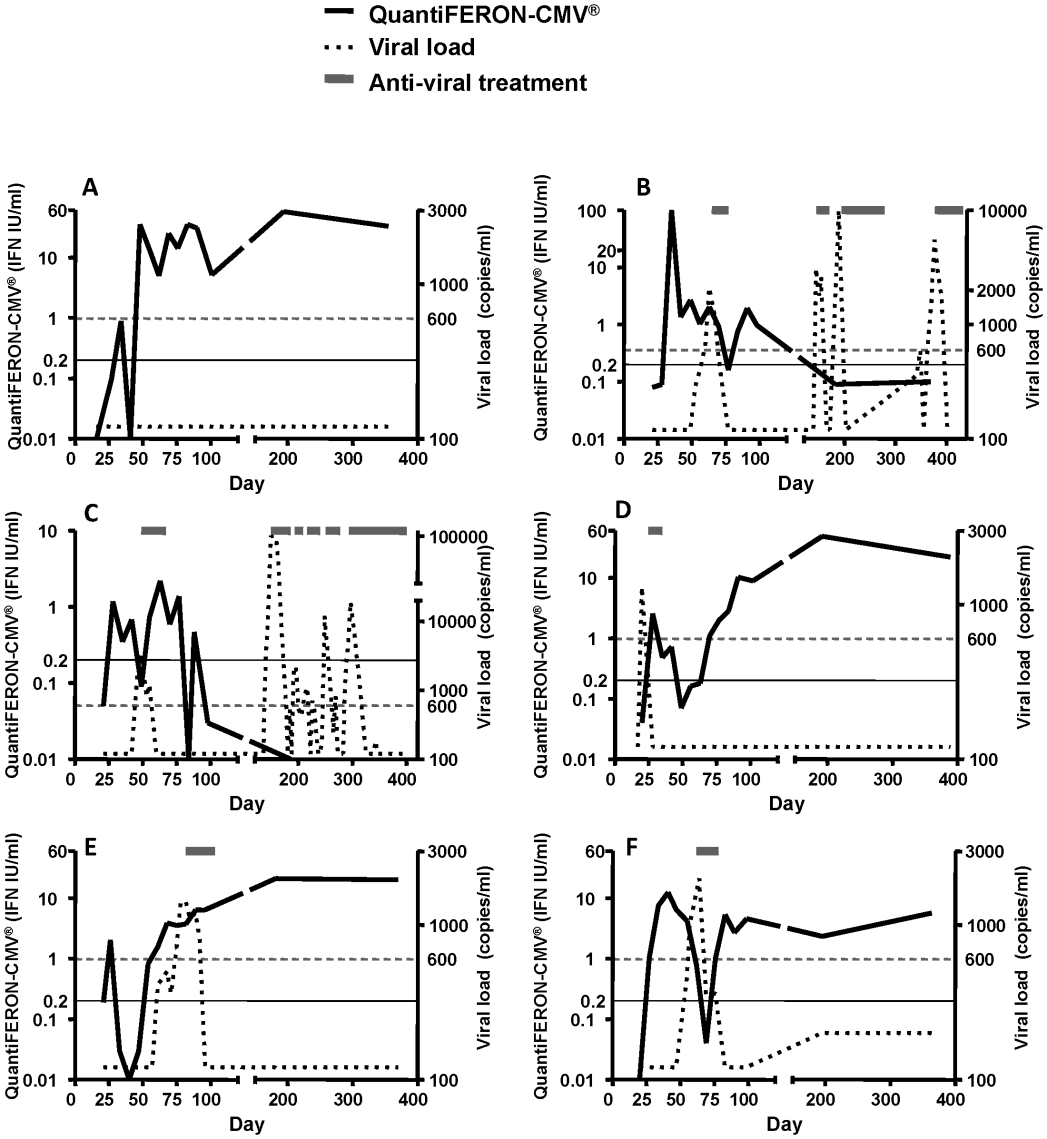
Individual patient plots showing the relationship between CMV-specific CD8+ T cell immune reconstitution and virus reactivation. QuantiFERON-CMV® readings are shown in black continuous lines; the threshold for positive QuantiFERON-CMV® reading (0.2 IU/ml) is indicated by a light continuous line. CMV viral loads are shown in dotted lines; the threshold for pre-emptive antiviral treatment (600 copies/ml) is indicated by a light dotted line. Anti-viral treatments are indicated by grey bars.

To better understand the temporal relationship between CMV reactivation and immune reconstitution, the data log from the diagnostic laboratory was reviewed to obtain all episodes of CMV DNAemia, including results that were below the reporting threshold of 600 copies/ml. Representative data from six patients are presented in Fig. 3. This analysis revealed that the reconstitution of CMV-specific T cell immunity often occurred around the time of CMV reactivation (Fig. 3). In some patients, no viral reactivation was observed if CMV-specific T cell immunity was reconstituted and remained stably reconstituted throughout the follow up period (Fig. 3; Panel A). In patients who were unable to stably establish CMV-specific T cell immunity, recurrent viral reactivation was observed, requiring multiple courses of anti-viral treatment (Fig. 3; Panel B & C). In other patients, single episodes of overt CMV reactivation (≥600 copies/ml) were observed within 100 days of transplant, requiring anti-viral therapy (Panel D–F). These episodes of viral reactivation were observed either before the establishment of anti-viral immunity (Panel D) or during the transient reduction/loss in antigen-specific T cell responses (Panel F). Following the resolution of CMV reactivation, stable CMV-specific T cell immunity was established and there were no further episodes of viral reactivation (Fig. 3; Panel D–F).

#### Immune reconstitution and virus control following reactivation

**Figure 4:**
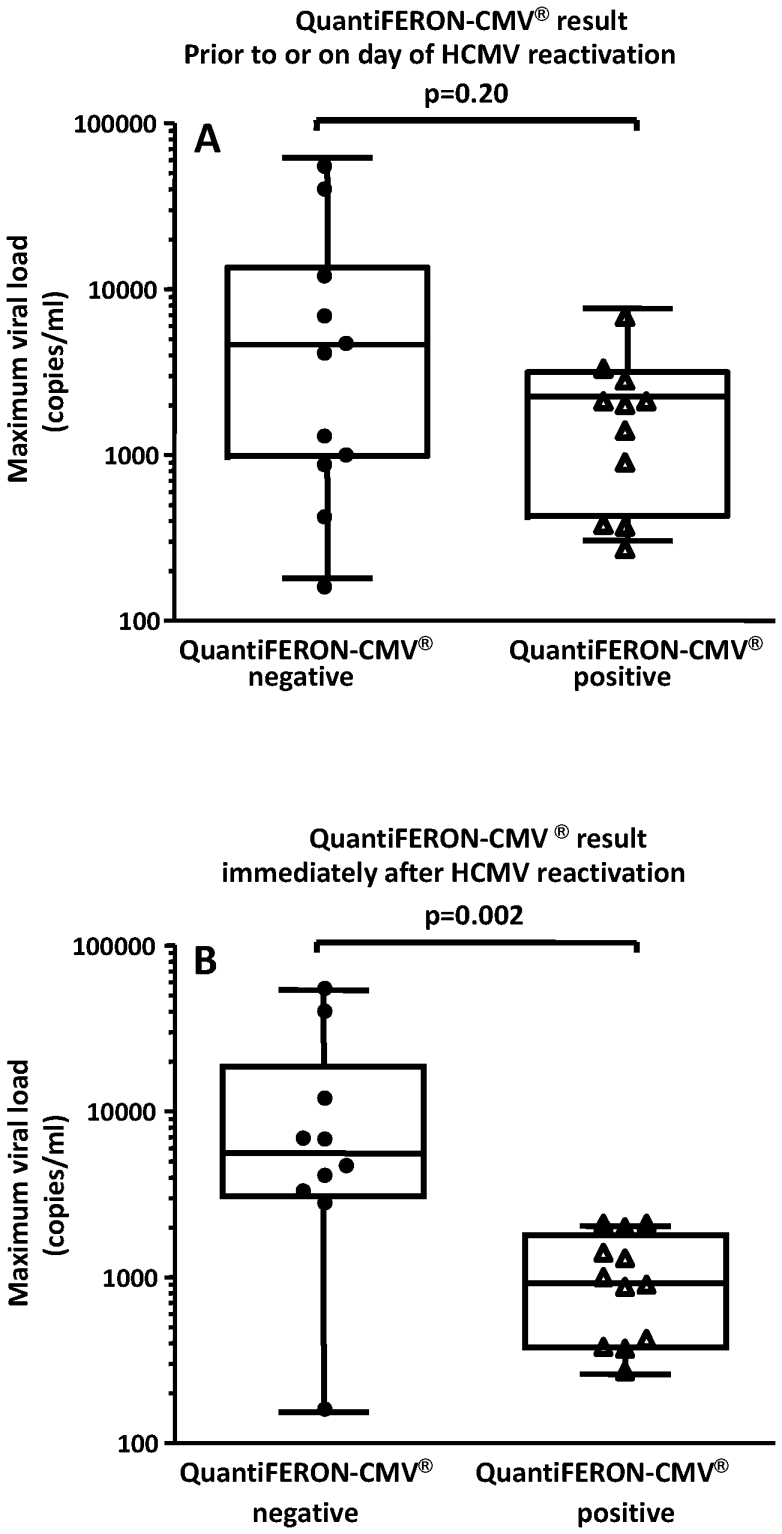
Effect of QuantiFERON-CMV® results on the maximum viral load. Patients were grouped according to QuantiFERON-CMV® results (a) just prior to or on the day of first virus reactivation and (b) results immediately after evidence of virus reactivation (4.0±2.4 days, mean ± SD; range 1 to 8 days). Box and whisker plots show the upper and lower quartiles (box) with median value indicated as line in the box, while whiskers show maximum and minimum values.

QuantiFERON-CMV® results were available in the week (mean 3.3 days, range 0–6) leading up to CMV DNAemia in 22 patients: results were positive in 11 and negative in 11. On serial testing a week later (mean 7.3; range 6 to 9 days), the QuantiFERON-CMV® result converted from negative to positive in 4 patients and from positive to negative in 3 patients. Using the maximum viral load as a measure of viremia control, HSCT patients who were QuantiFERON-CMV® positive prior to viral reactivation showed a median viral load of 1347 copies/ml, while the median viral load in QuantiFERON-CMV® negative patients was 3116 copies/ml, however, this difference was not statistically significant (p = 0.20, *t* test) (Fig. 4A). Assessment of immune responses with the QuantiFERON-CMV® assay immediately (4.0±2.4 days, mean ± SD; range 1 to 8 days) after the viral reactivation showed that the median CMV load in QuantiFERON-CMV® positive HSCT recipients was significantly lower (875 copies/ml), when compared to the patients who showed negative response in the QuantiFERON-CMV assay (5685 copies/ml) (p = 0.002, *t* test) (Fig. 4B). Of note, 5 out of 12 patients with CMV-specific immunity cleared the viraemia spontaneously as compared to only 1 out of 10 patients without CMV-specific immunity (p = 0.16; Fisher's exact test). Taken together these analyses indicate that the development of CMV-specific immunity was essential for viral control and a failure to reconstitute CMV-specific immunity was associated with higher peak viral loads.

## Discussion

In this study, we formally assessed the clinical utility of a whole blood QuantiFERON-CMV® assay as an immune monitoring tool to predict CMV-associated complications in allogeneic HSCT recipients. The QuantiFERON-CMV® assay is based on the observation that anti-viral T cell immunity is a key determinant of the risk of CMV reactivation in allogeneic HSCT. Patients with delayed CMV-specific immune reconstitution have a higher incidence of recurrent or complicated CMV reactivation [6], [8], [11], [23]. Over the last decade, a number of novel technologies (e.g. ELISPOT, MHC-peptide tetramers and intracellular cytokine assay) have been developed which have revolutionized *ex vivo* monitoring of human immune responses. Despite extensive preclinical evaluation, most of these technologies are yet to be adopted in formal clinical settings. The QuantiFERON-CMV® assay is the only functional T cell monitoring assay that is licensed for use as an *in vitro* diagnostic assay for transplant patients. This assay measures IFN-γ secretion by CMV-specific CD8^+^ T cells in whole blood following stimulation by a pool of previously defined MHC class I-restricted peptide epitopes from multiple CMV antigens including pp65, IE-1, IE-2, pp50 and gB. In this study, a total of 41 evaluable allogeneic HSCT recipients underwent weekly immunological monitoring using QuantiFERON-CMV® assay and data from this assay was correlated with viral load and other clinical indicators. A number of conclusions were drawn from these studies.

Firstly, the reconstitution of CMV-specific T cell immunity as assessed with QuantiFERON-CMV® assay was highly dynamic at the early stages post HSCT transplant. Although a large proportion of HSCT recipients showed single positive results for QuantiFERON-CMV® assay during the early stages post transplant, subsequent testing often showed one or more negative or indeterminate results. Long-term follow up showed that many HSCT recipients attained stable immune reconstitution (median time point: 59 days post-transplant) and importantly, a lower proportion of these patients had CMV reactivation as compared to those who did not develop stable QuantiFERON-CMV® positive results. Longitudinal analysis of CMV-specific T cell responses in individual patients showed that the acquisition of stable immune reconstitution was coincident with reduced viral reactivation episodes, while more frequent CMV reactivations were observed in HSCT recipients whose QuantiFERON-CMV® positivity was not sustained.

Secondly, we also observed an intricate relationship between the QuantiFERON-CMV® assay readout and virus control following an initial episode of viral reactivation. A clear and significant impact of immune reconstitution was observed when the QuantiFERON-CMV® assay was carried out in the first week following viral reactivation. Patients who were QuantiFERON-CMV® positive in the first week following viral reactivation had a maximal viral load which was 6.5 fold lower than patients who were QuantiFERON-CMV® negative. Furthermore, 42% of the QuantiFERON-CMV® positive HSCT recipients cleared the viraemia spontaneously while only 10% of patients did so without CMV-specific T cell immunity. These observations further emphasized the importance of anti-viral T cell immunity for viral control and a failure to reconstitute CMV-specific immunity was associated with higher peak viral loads.

Finally, although this study provides strong support for the potential application of serial monitoring of CD8+ T cells using the QuantiFERON-CMV® assay in the allogeneic HSCT setting, further studies involving larger groups of patients should be carried out to provide more conclusive data on the use of this assay as a prognostic tool. There are a number of potential limitations of the current study which needs to be addressed in future studies. Although the QuantiFERON-CMV® assay is primarily designed to assess the CD8+ T cell responses, it is important to mention here that a number of groups have highlighted the importance of both CD8+ and CD4+ T cells in controlling CMV infection and disease in both allogeneic HSCT and solid organ transplant recipients. Indeed, studies carried out by Einsele and colleagues have shown that the infusion of low numbers of CMV-specific CD4+ T-cells was coincident with a rapid antiviral effect in allogeneic HSCT recipients [24]. Thus it is possible that the inclusion of CD4+ T cell epitopes in the QuantiFERON-CMV® assay may improve its sensitivity and specificity as a prognostic tool. Furthermore, in the current study we were unable to evaluate the predictive value of QuantiFERON-CMV® assay for CMV disease since very low number of patients progressed to virus-associated complications due to the routine use of pre-emptive antiviral therapy. In spite of these limitations, the current study provides an important platform for a more comprehensive evaluation of QuantiFERON-CMV® assay in a multicentre setting to firmly establish the diagnostic potential of this novel assay, particularly in higher risk patients, such as those with moderate to severe GVHD, as shown here and by others [25]. The fluctuation in QuantiFERON-CMV® results seen in this study is possibly a reflection of changes in the depth of pharmacological immunosuppression. False positives and false negatives would be highly unlikely given that in the initial assay validation, 97% of CMV-seropositive individuals were QuantiFERON-CMV® positive and all CMV-seronegative individuals were QuantiFERON-CMV®negative (Walker et al). Furthermore, there was good correlation between QuantiFERON-CMV® results and IFN-γ EliSPOT in solid organ transplant patients [15]. It is possible that allogeneic stem cell transplantation and its attendant immunosuppressive therapy could impact on the cytokine secretion profile and that the sensitivity could be enhanced with the inclusion of other cytokines, such as tumour necrosis factor (TNF) and macrophage inflammatory protein-1b (MIP1b), but this will add to the complexity and cost without necessarily any added clinical benefit. It is important to emphasize that QuantiFERON-CMV® assay is a technically simple whole blood functional assay that has similar functional characteristics to other, more demanding, assays being investigated for the immunological monitoring of allogeneic HSCT patients. We propose that QuantiFERON-CMV® based longitudinal immunological monitoring either in the week after the onset of a first reactivation or after two months of HSCT can be considered for identifying patients at risk of complicated reactivation.
